# Utilization of CT Pulmonary Angiography in Suspected Pulmonary Embolism in a Major Urban Emergency Department

**DOI:** 10.1155/2013/915213

**Published:** 2013-08-29

**Authors:** Adil Shujaat, Janet M. Shapiro, Edward Eden

**Affiliations:** ^1^Division of Pulmonary and Critical Care Medicine, College of Medicine at Jacksonville, University of Florida, Jacksonville, FL, USA; ^2^University of Florida, Shands Clinical Center, 655 West 8th Street, Suite 7-088, Jacksonville, FL 32209, USA; ^3^Division of Pulmonary and Critical Care Medicine, St. Luke's and Roosevelt Hospitals of Columbia University, New York, NY, USA

## Abstract

*Objectives*. We conducted a study to answer 3 questions: (1) is CT pulmonary angiography (CTPA) overutilized in suspected pulmonary embolism (PE)? (2) What alternative diagnoses are provided by CTPA? (3) Can CTPA be used to evaluate right ventricular dilatation (RVD)? *Methods*. We retrospectively reviewed the clinical information of 231 consecutive emergency department patients who underwent CTPA for suspected PE over a one-year period. *Results*. The mean age of our patients was 53 years, and 58.4% were women. The prevalence of PE was 20.7%. Among the 136 patients with low clinical probability of PE, a d-dimer test was done in 54.4%, and it was normal in 24.3%; none of these patients had PE. The most common alternative findings on CTPA were emphysema (7.6%), pneumonia (7%), atelectasis (5.5%), bronchiectasis (3.8%), and congestive heart failure (3.3%). The sensitivity and negative predictive value of CTPA for (RVD) was 92% and 80%, respectively. *Conclusions*. PE could have been excluded without CTPA in ~1 out of 4 patients with low clinical probability of PE, if a formal assessment of probability and d-dimer test had been done. In patients without PE, CTPA did not provide an alternative diagnosis in 65%. In patients with PE, CTPA showed the potential to evaluate RVD.

## 1. Introduction

The paradox in the diagnosis of pulmonary embolism (PE) is that it tends to be both underdiagnosed and overinvestigated. The prevalence of PE-varies from 10% to 25% in different studies [1–5]. The vast majority (94%) of PE related deaths are because of a failure of diagnosis [6]. The consequences of missing the diagnosis and the ease of recalling prior serious cases may lead to an overestimation of the probability of PE and lower the threshold for initiating a cascade of diagnostic testing, a phenomenon described as the availability heuristics in cognitive psychology [7, 8]. The widespread round-the-clock availability, excellent accuracy [9, 10] of CT pulmonary angiography (CTPA), and ability to provide an alternative diagnosis [11, 12] may further lower the threshold for performing this imaging study and result in its overuse. On the other hand, outcome studies using clinical prediction rules to refine diagnostic certainty have shown that PE can be safely excluded in patients with low clinical probability and normal d-dimer levels without an imaging study [1, 2, 5]. However, the impact of such evidence-based strategies on actual clinical practice is not known. In this era of evidence-based decision making and cost-effective utilization of resources, it is imperative to diagnose and risk-stratify emergency department (ED) patients with pulmonary embolism in a more objective manner. We conducted a study to determine if the utilization of CTPA in suspected PE could be refined. We sought to answer these three questions: (1) is CTPA overutilized? (2) What alternative or incidental diagnoses are provided by CTPA? (3) Can CTPA be used to evaluate right ventricular dilatation (RVD)?

## 2. Methods

We retrospectively reviewed the clinical information of 231 consecutive ED patients who were suspected of PE and underwent a CTPA during the one-year period, January 2005 to December 2005 at St. Luke's Hospital which is a university affiliated hospital in New York City. The study was approved by the Institutional Review Board of St. Luke's and Roosevelt Hospitals. 

We collected information on age, gender, presenting complaints, PE risk factors, physical examination, chest radiographs, electrocardiogram, arterial blood gas, d-dimer levels, CTPA, and echocardiography. The immunoturbidimetric STA-Lia test was used for plasma d-dimer. The VITRO ECi immunoassay was used for plasma troponin I. All CTPA studies were done on Toshiba's Aquilion MULTI (a 34-row detector CT scanner). The diagnosis of PE was excluded if CTPA did not show any evidence of PE.

One investigator retrospectively applied Wells' simplified clinical prediction model [13] without knowledge of results of the d-dimer levels and the CTPA (Table 1). One investigator reviewed the CTPA of patients without PE for alternative or incidental findings. One investigator reviewed the CTPA of those with PE for evidence of right ventricular dilatation (RVD) without knowledge of the echocardiography results. RVD was defined as the short axis of the right ventricle is larger than that of the left ventricle when measured between the inner surface of the free wall and the surface of the interventricular septum on a single axial image where both appeared maximally distended [14].

## 3. Results

### 3.1. Clinical Characteristics of the Patients

The mean age of the patients was 53 years, and 58.4% were women. The most common presenting complaints were dyspnea, chest pain, or both. Only 2 patients had hemoptysis. The most common risk factors for PE were malignancy, a previous history of deep vein thrombosis or PE, and immobilization or surgery (Table 2).

### 3.2. Probability Groups and Prevalence of PE

Of the 231 patients suspected of PE in which a CTPA was performed, 48 (20.7%) had evidence of PE. The prevalence of PE was 7.3%, 42.2%, and 100% in the low, moderate, and high probability groups, respectively (Tables 3 and 4). 

### 3.3. Accuracy of d-Dimer

The sensitivity, specificity, negative predictive value and positive predictive value, of d-dimer were 90.4%, 34.5%, 94.8%, and 21.3%, respectively. 

### 3.4. Alternative Findings on CTPA

The CTPA did not reveal an alternative finding in the majority (65%) of the patients. The most common alternative findings on CTPA were emphysema (7.6%), pneumonia (7.1%), and atelectasis (5.5%) (Table 5). 

### 3.5. Prevalence of RVD

Of the 23 patients with PE who had echocardiography, 12 (52%) had evidence of RVD. The prevalence of RVD on CTPA was 55% (26/47 patients).

### 3.6. Accuracy of CTPA for RVD

The sensitivity, specificity, negative predictive value, and positive predictive value of CTPA for RVD were 90.9%, 44.4%, 80%, and 66.6%, respectively.

### 3.7. Utilization of Diagnostic Studies

Of the 231 patients who were suspected of PE and underwent CTPA, 136 (58.8%) had a low clinical probability of PE. Of these patients, only 74 (54.4%) had a d-dimer sample sent as part of the diagnostic evaluation for PE. Of these 74 patients, 18 (24.3%) had normal d-dimer levels (<0.58 *μ*g/mL). None of these patients had evidence of PE on CTPA.

If a clinical probability of PE had been assigned to all the ED patients suspected of PE and all the patients with a low clinical probability of PE had had a d-dimer sample sent, approximately one out of four CTPAs could have been avoided.

## 4. Discussion

The prevalence of PE in our study (20%) is comparable to that reported in the medical literature (10–25%) [1–5]. Similarly, the proportion of patients determined to have a low clinical probability (52%) is comparable to that cited in the literature (53–58%) [3]. However, a d-dimer sample was sent in only 54% of the patients with a low clinical probability, and it was normal in only 24% of these patients. Outcome studies have shown that PE can be excluded in patients with a low clinical probability and a low d-dimer result without the need for a CTPA [1, 2, 5]. Our study supports the hypothesis that CTPA is being overused and d-dimer underutilized in the diagnostic evaluation of ED patients suspected of PE. It shows that in a major urban ED like ours, where no clinical practice guideline for evaluation of PE was in place, if a clinical prediction model is used to assign a probability to ED patients suspected of PE and a d-dimer sample is sent in all the patients with a low clinical probability of PE, a CTPA can be avoided in approximately one-quarter of such patients. 

An accurate determination of clinical probability of PE is important because the interpretation of diagnostic studies depends upon this probability. The presenting symptoms and signs of PE are nonspecific, and only 10–25% of the patients suspected of PE actually turn out to have it [1–5]. The accuracy and interobserver reliability of an empiric clinical probability assessment of PE by overall impression are poor [15] and inversely proportional to clinical experience [15, 16]. Recently two derived clinical prediction models have been externally validated and evaluated in outcome studies [1–5]. The Canadian Wells' simplified clinical prediction model [13] has the advantage over the Geneva model [17] of having been studied in both inpatients and outpatients and of having a moderate to substantial inter-observer agreement. 

The sensitivity and negative predictive value of STA-Lia test d-dimer in our study were 90.4% and 94.8%, which are comparable to the sensitivity and negative predictive value of 98% and 97% cited in studies evaluating this assay [18, 19]. 

CTPA has replaced ventilation-perfusion scan as the imaging test of choice in patients suspected of PE. Outcome studies have shown it to be comparable to the “gold standard” conventional pulmonary angiography, which is seldom performed [10, 11]. It is available round the clock and also carries the potential advantage of providing an alternative diagnosis in those who turn out to not have PE. Few studies have examined the frequency and validity of alternative diagnoses in those who turn out to not have PE [12, 13]. Our study shows that CTPA revealed alternative findings in only 35% of such patients. The most common alternative findings were emphysema (7.6%), pneumonia (7.1%), atelectasis (5.5%), bronchiectasis (3.8%), air-trapping (3.3%), and pulmonary edema (3.3%). 

CTPA is not without its drawbacks. There is a finite risk of general adverse reactions to iodinated contrast dye. The incidence of acute general adverse reactions varies and is 15% for mild reactions (nausea, vomiting, limited urticaria, and pallor), 1-2% for moderate ones (severe vomiting, extensive urticaria, laryngeal edema, and dyspnea), 0.2% for severe ones (pulmonary edema, arrhythmia, cardiac arrest, and circulatory collapse) [20]. There is also a risk of contrast-induced nephropathy that varies from 1% in patients with normal renal function to 50% in those with diabetic nephropathy [20], especially in patients congestive heart failure and with cor pulmonale who are on diuretics and are at high risk for PE. In contrast, when d-dimer is normal the probability of PE is low, and CTPA is not performed, the risk of PE during 3-month followup is only 0.2% [1]. Similarly, when d-dimer is normal PE is unlikely, and CTPA is not performed, the risk of nonfatal venous thromboembolism is only 0.5% [5].

More importantly, there is an underappreciated risk of radiation exposure from CTPA, which cannot be ignored in young women smokers on oral contraceptive pills and pregnant women who comprise a high-risk group for PE. Moreover, 60% of CTPA studies performed over a 2-year period at one institution were on women [21]. Interestingly, a similar proportion of CTPA studies done in our study were on women. CTPA delivers a minimum radiation dose of 2.0 rad (20 mGy) to each breast in an average-sized woman. By contrast, ventilation-perfusion scan delivers a dose of 0.28 mGy [21]. A 20-year-old woman receiving a dose of 40 mGy from a single CTPA study has been estimated to be at 68% greater risk for breast cancer by age 35 years than a 20-year-old woman without such exposure [22]. Because the thyroid, breast, and lungs are among the most cancer-susceptible organs in the body and are included in chest CT scan, the large scale of use may have epidemiologic significance. Estimates suggest that 6,800 future cancers may be attributable to chest CT scans performed in 2007 alone [23] and that 0.7% to 2% of all future cancers in the United States may be caused by radiation from CT scan [24, 25]. Nevertheless, CTPA is preferred over ventilation-perfusion scan for suspected PE in pregnant patients when venous ultrasonography of the legs is unrevealing. Although CTPA delivers a higher dose of radiation to the mother, it delivers a lower dose to the fetus than a ventilation-perfusion scan [26]. Lastly, use of CTPA for suspected PE without a formal assessment of probability of PE and d-dimer levels is not a cost-effective approach. Lee et al. performed a cost-effectiveness analysis of diagnostic strategies in suspected PE and showed that the strategy combining clinical probability assessment, highly sensitive rapid d-dimer assay (97% sensitivity), and multidetector CTPA had the lowest cost per life saved [27]. Moreover, the cost per life saved was $1258 when the clinical probability of PE was low compared to $3122 and $5496 when it was intermediate and high, respectively [27].

PE is a heterogeneous disorder that carries a highly variable mortality depending on its presentation and the patient's underlying cardiopulmonary status. Mortality varies from 1.5% in the case of a hemodynamically stable patient treated with anticoagulation alone [6] to almost certain death in the case of a cardiopulmonary arrest. There is a select group of hemodynamically stable PE patients with evidence of right ventricular dysfunction that need to be identified. Ten percent of such patients can decompensate and half of these can die [28]. Recognition of this group of patients at risk of hemodynamic deterioration is important in order to transfer them to the medical intensive care unit for closer monitoring and consideration of thrombolysis if necessary. Reliance on the availability and expertise of echocardiography for identifying such patients especially in the after-hours can lead to delay in triage and result in crowding in the ED. A few studies have suggested that CTPA can be used to evaluate RVD in PE patients [14, 29, 30]. Although we did not evaluate the outcome of patients who turned out to have PE, our study suggests that CTPA has the potential to evaluate RVD in such patients. The prevalence of RVD in the patients who turned out to have PE was 52% (12/23 patients) on echocardiography and 55% (26/47 patients) on CTPA. This is not much different from the ~30 to 55% prevalence cited in the literature [21, 31–33]. The high sensitivity (91.6%) of CTPA for RVD in our study is similar to that reported in a retrospective study of 110 consecutive patients suspected of PE. However, our study shows a lower specificity (44.4%) compared to that study (100%) [14]. This could be for a number of reasons: firstly, Lim et al. studied only patients with acute massive pulmonary embolism, whereas we studied all the patients with PE. Secondly, there were a significant number (44%) of technically difficult echocardiographic studies in our patients. Thirdly, 36% of the echocardiographic studies were done more than 24 hours after the CTPA by which time RVD may have resolved. 

## 5. Limitations

Our study is not without limitations. The retrospective nature of our study made it difficult to calculate the Wells' simplified model score in 16 (~7%) of the 231 patients because of missing data. However, none of these patients was diagnosed with PE on the CTPA. We did not follow up on the patients in whom PE was excluded. However, outcome studies have shown that the clinical validity of using a CTPA to rule out PE is similar to that reported for conventional pulmonary angiography. Although our study shows that CTPA revealed alternative findings in only 35% of such patients, we did not correlate the findings with the actual alternative diagnoses given to these patients. Our study suggests that CTPA has the potential to evaluate right ventricular dilatation. However, right ventricular *dilatation* alone may not reflect right ventricular *dysfunction*, and studies have defined right ventricular *dysfunction* on echocardiography as right ventricular hypokinesia or using composite criteria that included right ventricular *dilatation* with a threshold for right ventricular end-diastolic diameter/left ventricular end-diastolic diameter ratio of 0.6–1 [34, 35]. More importantly, we did not follow up the outcome of patients with RVD to determine the direct role of CTPA in risk-stratification. Lastly, we did not evaluate those ED patients who were suspected of PE but did not undergo a CTPA.

## 6. Conclusions

The choice of clinical prediction model is not as important as the fundamental principle of using such a model to accurately determine the clinical probability in each patient with suspected PE because the interpretation of diagnostic tests depends upon an accurate assessment of clinical probability. Since PE can be safely excluded without a CTPA in patients with a low clinical probability and a normal d-dimer level, application of a prediction model and use of d-dimer can refine diagnostic certainty and reduce the disproportionate number of CTPAs being done in such patients. Moreover, contrary to popular belief, CTPA did not reveal an alternative finding in 65% of the patients without PE. The usefulness of CTPA to evaluate RVD can potentially risk-stratify ED patients with PE, especially in the after-hours when availability of echocardiography is limited.

## Figures and Tables

**Table 1:** Wells' simplified clinical prediction model.

Parameter	Points
Clinical symptoms or signs of deep vein thrombosis (DVT)	3
Heart rate > 100 beats per minute	1.5
Immobilization (for 3 or more days) or surgery in the last 4 weeks	1.5
Previous history of DVT or PE	1.5
Hemoptysis	1
Malignancy (diagnosed in the last 6 months, under active or palliative treatment)	1
Alternative diagnosis less likely than PE (based on presenting history, physical examination, CXR, EKG, and ABG)	3

Clinical probability of PE	Score

Low	<2
Moderate	2–6
High	>6

**Table 2:** Clinical characteristics of 231 patients suspected of having a PE in whom CTPA was ordered.

Age in years (mean)	53	
	*N *	%
Female gender	135	58.4
Pregnant	3	1.3
Dyspnea	73	31.6
Chest pain	58	25
Dyspnea and chest pain	22	9.5
Syncope	9	3.8
Near-syncope/dizziness	7	3
Leg pain/swelling	14	6
Heart rate > 100 beats/minute	78	33.7
Immobilization or surgery	12	5.2
Previous DVT or PE	19	8.2
Hemoptysis	2	0.86
Malignancy	24	10.3

**Table 3:** Clinical probability groups based on Wells' simplified prediction model.

Probability group	*n*/*N*	%
Low clinical probability	136/231	58.8
Moderate clinical probability	71/231	30.7
High clinical probability	8/231	3.5
Unknown	16/231	6.9

**Table 4:** Probability groups and prevalence of PE.

Prevalence of PE	*n*/*N*	%
Overall	48/231	20.7
Low clinical probability group	10/136	7.3
Moderate clinical probability group	30/71	42.2
High clinical probability group	8/8	100
Unknown	0/16	0

**Table 5:** Most common alternative or incidental findings on CTPA of patients without PE (*N* = 183).

	*N*	%
Emphysema	14	7.6
Pneumonia	13	7.1
Atelectasis	10	5.5
Bronchiectasis	7	3.8
Air trapping	6	3.3
Congestive heart failure	6	3.3
Pleural effusion	5	2.7
New pulmonary nodule/mass	3	1.6
No alternative finding	119	65

## Abbreviations

CT: Computed tomography
CTPA: CT pulmonary angiography
PE: Pulmonary embolism
RVD: Right ventricular dilatation
ED: Emergency department.
